# Supplementary material for: The interactome of intact mitochondria by cross-linking mass spectrometry provides evidence for coexisting respiratory supercomplexes
Source: Mol Cell Proteomics. 2017 Dec 8;17(2):216–32. doi: 10.1074/mcp.RA117.000470 (PMC5795388; doi:10.1074/mcp.RA117.000470)
Supplement: Supplemental Data [file supp_RA117.000470_133922_0_supp_23978_fzffwf.zip › spectra_annotation/mito_spectra_annotation/124-1-4-1-9-1.pdf]

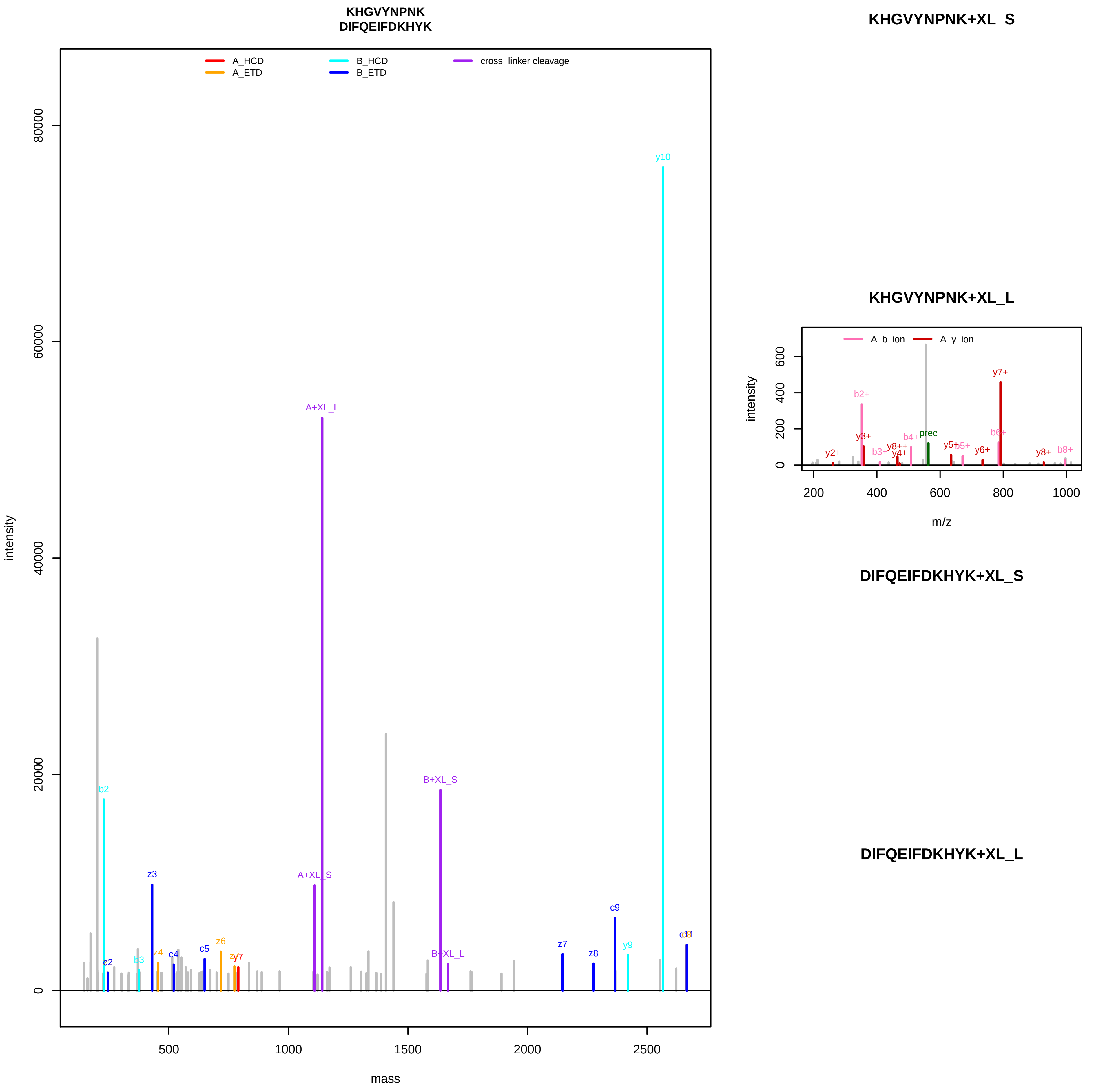
KHGVYNPNK
DIFQEIFDKHYK
A_HCD
A_ETD
B_HCD
B_ETD
cross−linker cleavage
intensity
mass
0
20000
40000
60000
80000
500
1000
1500
2000
2500
y10
A+XL_L
B+XL_S
A+XL_S
B+XL_L
b2
b3
c2
z3
z4
c4
c5
z6
z7
y7
z7
z8
c9
y9
c11
KHGVYNPNK+XL_S
KHGVYNPNK+XL_L
A_b_ion
A_y_ion
intensity
m/z
0
200
400
600
200
400
600
800
1000
b2+
y3+
y7+
y2+
b3+
y8++
y4+
b4+
prec
y5+
b5+
y6+
b6+
y8+
b8+
DIFQEIFDKHYK+XL_S
DIFQEIFDKHYK+XL_L
